# Large-scale metabolomic profiling and incident non-alcoholic fatty liver disease

**DOI:** 10.1016/j.isci.2023.107127

**Published:** 2023-06-14

**Authors:** Eloi Gagnon, Hasanga D. Manikpurage, Patricia L. Mitchell, Arnaud Girard, Émilie Gobeil, Jérôme Bourgault, Frédéric Bégin, André Marette, Sébastien Thériault, Benoit J. Arsenault

**Affiliations:** 1 Centre de Recherche de L’Institut Universitaire de Cardiologie et de Pneumologie de Québec, Québec (QC), Canada; 2 Department of Medicine, Faculty of Medicine, Université Laval, Québec (QC), Canada; 3 Department of Molecular Biology, Medical Biochemistry and Pathology, Faculty of Medicine, Université Laval, Québec (QC), Canada

**Keywords:** Hepatology, Pathophysiology, Human metabolism, Genetics

## Abstract

Non-alcoholic fatty liver disease (NAFLD) is a highly prevalent disease with no specific drug therapy. High-throughput metabolomics present an unprecedented opportunity to identify biomarkers and potentially causal risk factors for NAFLD. Here, we determined the impact of 21 circulating metabolites, 17 lipids, and 132 lipoprotein particle characteristics on NAFLD combining prospective observational and two-sample Mendelian randomization (MR) analyses in 121,032 UK Biobank participants. We identified several metabolic factors associated with NAFLD risk in observational and MR analyses including triglyceride-rich and high-density lipoprotein particles composition, as well as the ratio of polyunsaturated fatty acids to total fatty acids. This study, is one of the largest to investigate incident NAFLD, provides concordant observational and genetic evidence that therapies aimed at reducing circulating triglycerides and increasing large HDL particles, as well as interventions aimed at increasing polyunsaturated fatty acid content may warrant further investigation into NAFLD prevention and treatment.

## Highlights

• Triglycerides increase non-alcoholic fatty liver disease (NAFLD) risk.
• High-density lipoprotein particles decrease NAFLD risk.
• The polyunsaturated/fatty acid ratio decreases NAFLD risk.
• Branched-chained amino acids are not causally linked with NAFLD.

## Graphical abstract

**Figure:**
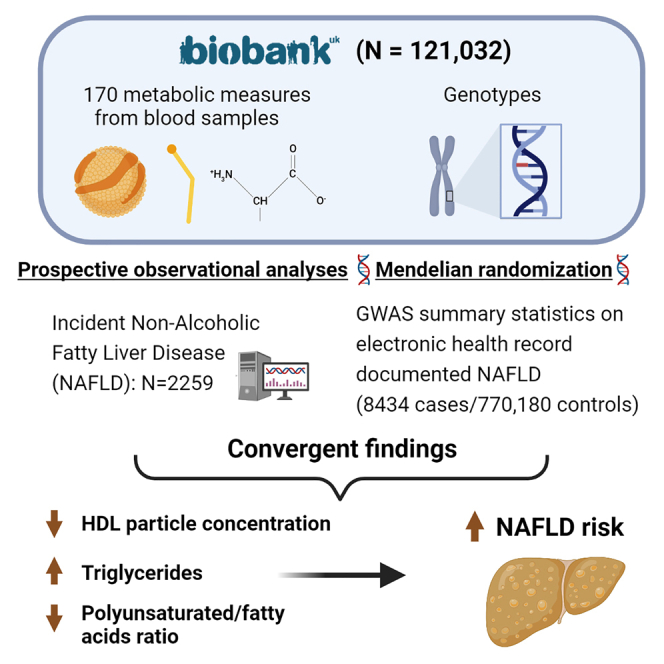

## Introduction

Worldwide, up to 25% of the population could be affected by non-alcoholic fatty liver disease (NAFLD). NAFLD is one of the leading causes of serious liver complications, such as steatohepatitis (NASH), fibrosis, cirrhosis, liver failure, and hepatocellular carcinoma.^1^^,^^2^ The prevalence of NAFLD and its associated sequelae has been increasing for the past decades, posing a serious threat to global population health.^3^ NAFLD is characterized by an accumulation of lipids within hepatocytes (> 5% of hepatocyte mass). It is associated with abdominal adiposity and disturbances in hepatocyte lipid handling.^4^ Therapeutic options are often limited to lifestyle changes, and effective and specific drug treatment for NAFLD is still an important unmet medical need.

In recent years, the development of high-throughput metabolomic platforms has provided unique opportunities to test the association of hundreds of metabolic measures with complex diseases such as NAFLD. Previous metabolomic and lipidomic clinical studies have identified biomarkers associated with NAFLD and NASH.^5^ Metabolomic observational studies have found that the blood level of several amino acids is elevated in patients with NAFLD.^6^ Lipidomic observational studies reported plasma lipid abnormalities in NAFLD such as higher levels of VLDL (very low-density lipoproteins) and LDL (low-density lipoproteins), as well as lower levels of HDL (high-density lipoproteins) cholesterol.^7^^,^^8^ However, these studies had small sample sizes and had cross-sectional designs so reverse causality and confounding cannot be excluded. Whether these metabolic biomarkers are simple correlates or causal drivers of NAFLD remains unclear. Consequently, it is unknown whether therapies designed to alter their blood levels could prevent the onset of NAFLD.

Finding effective therapies for NAFLD has been hampered by the difficulty of finding modifiable causal targets. NAFLD is diagnosed histologically through a biopsy, which is invasive and carries some risk to patients, or with imaging, which is costly, limiting the ability of large-scale randomized control trial to assess drug target efficacy. Novel and innovative approaches for drug target prioritization such as Mendelian randomization (MR) analyses have been used for cardiometabolic diseases such as coronary artery disease (CAD),^9^ but work using MR specifically for NAFLD remain sparse. MR leverages the power of large human genetic datasets to investigate causal links between a given risk factor (such as metabolites, lipids, lipoproteins, etc.) and a disease (such as NAFLD). MR assesses the phenotypic consequence of a lifelong genetic predisposition for altered levels of a given metabolic measure. MR provides valid causal inference when genetic instruments selected for MR analyses respect three core assumptions (relevance, exchangeability, and exclusion restriction).^10^ Abdominal adiposity, a known causal factor for both high lipid levels^11^ and NAFLD,^12^ could act as a confounding factor in the association of metabolic factors and NAFLD. Multivariable MR is a useful tool to adjust for measured confounders. Akin to a multivariable regression, multivariable MR assesses the direct effect of an exposure on an outcome adjusting for another variable that may confound the causal relation.^13^

**Figure 1:**
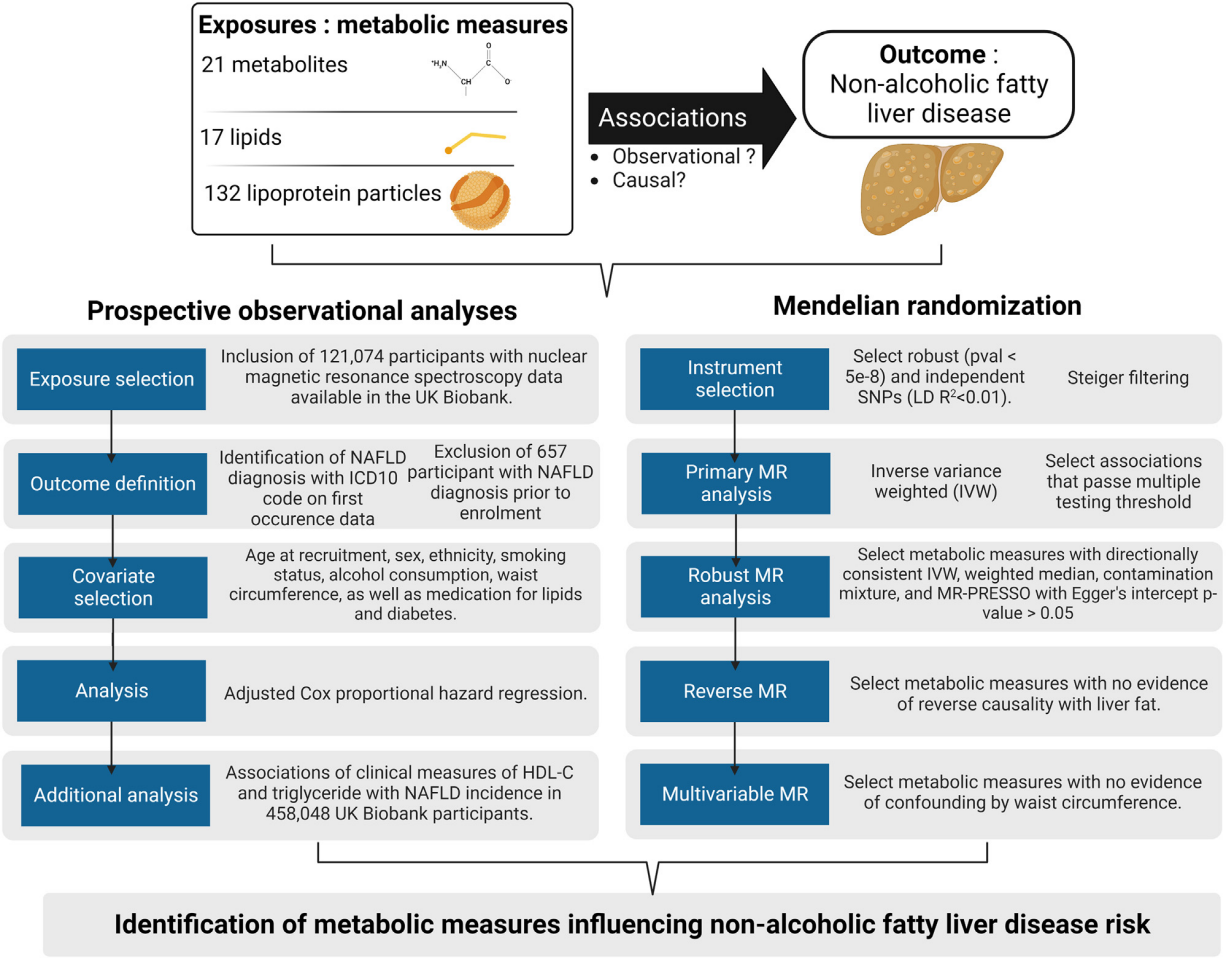
Schematic overview of the current study design The study included a prospective investigation in the UK Biobank, as well as a two-sample Mendelian randomization investigation into the impact of circulating metabolite with incident and prevalent non-alcoholic fatty liver disease, respectively. Related to STAR Methods.

Here, we performed a comprehensive series of prospective observational and MR analyses to determine the impact of circulating metabolites, lipids, and lipoprotein characteristics on NAFLD and to identify potential new targets for NAFLD prevention. We leveraged high-throughput metabolomic and lipidomic measures from 121,074 participants of the UK Biobank.^14^ We performed multivariable Cox proportional hazards regression investigating the association of 21 metabolites, 17 lipids, and 132 lipoprotein characteristics with incident NAFLD. We parallel these observational analyses with univariable and multivariable two-sample MR to investigate the potential causal contribution of these metabolic measures on NAFLD. An overview of our study design is presented in Figure 1.

## Results

### Observational analyses

The clinical characteristics of the study total sample and subsample with and without incident NAFLD are presented in Table 1. For observational analyses, we included all participants with nuclear magnetic resonance (NMR) data available (N = 121,731) and excluded all participants with an NAFLD diagnosis prior to enrollment (N = 699). Participants of the NMR subsample did not differ on key covariates from the total UKB sample (Table 1). We identified NAFLD time of diagnosis using first occurrence data (hepatic fibrosis, NASH, NAFLD, and other specified diseases of the liver without ICD10 exclusion code). During the median 12.6-year follow-up, 2259 participants had incident NAFLD.

**Table 1:** Baseline characteristics of the total UK Biobank sample and nuclear magnetic resonance spectroscopy subsample with and without non-alcoholic fatty liver disease related to STAR Methods

	Cases	Controls	Cases	Controls
N	12,304	490,107	2259	118,773
Age at enrollment, years (SD)	57.8 (7.8)	57.1 (8.1)	57.5 (7.8)	57.1 (8.1)
Male, n (%)	6072 (49.3)	223,014 (45.5)	1121 (49.6)	54,448 (45.8)
Waist circumference, cm (SD)	98.3 (14)	90.1 (13.4)	98.8 (13.4)	90.1 (13.4)
Triglycerides, mmol/L (SD)	2.2 (1.3)	1.7 (1)	2.2 (1.3)	1.7 (1)
HDL cholesterol, mmol/L (SD)	1.3 (0.4)	1.5 (0.4)	1.3 (0.4)	1.5 (0.4)
LDL cholesterol, mmol/L (SD)	3.5 (0.9)	3.6 (0.9)	3.4 (0.9)	3.6 (0.9)
Townsend deprivation index (SD)	−0.5 (3.4)	−1.3 (3.1)	−0.5 (3.4)	−1.4 (3.1)
Medications, n (%)	5444 (44.2)	132,778 (27.1)	1042 (46.1)	32,448 (27.3)
Smoking, n (%)	6513 (52.9)	219,474 (44.8)	1219 (54)	53,279 (44.9)
Alcohol consumption Likert scale (SD)	2.7 (1.7)	3.1 (1.5)	2.8 (1.6)	3.1 (1.5)
White ethnicity, n (%)	11,469 (93.2)	461,144 (94.1)	2101 (93)	112,172 (94.4)

Continuous variables are presented with mean values ± SD. Categorical variables are presented as n (%).

**Figure 2:**
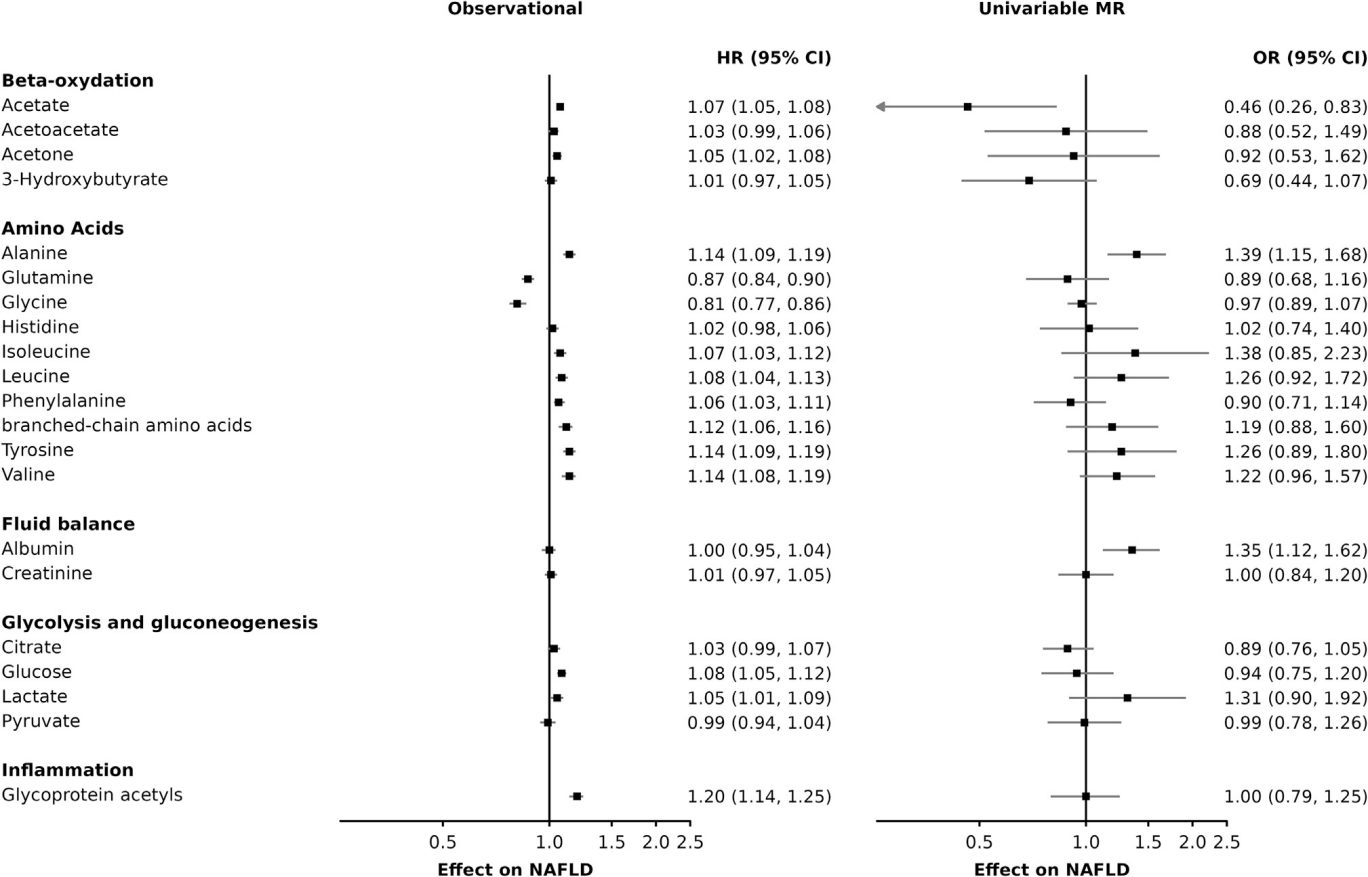
Effect of 1 standard deviation increase in metabolite concentrations on non-alcoholic fatty liver disease risk using observational and Mendelian randomization analyses Related to STAR Methods.

**Figure 3:**
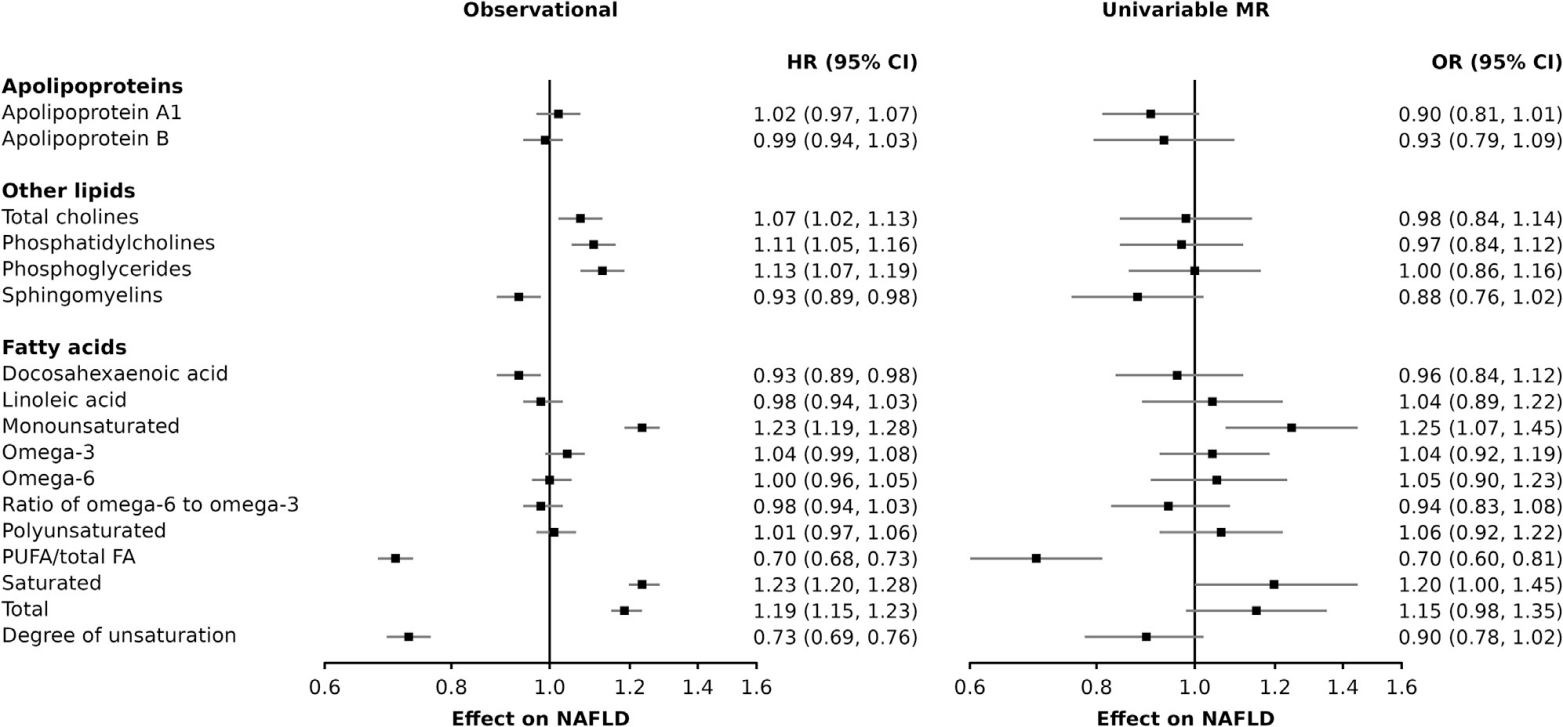
Effect of 1 standard deviation increase in lipid concentrations on non-alcoholic fatty liver disease risk using observational and Mendelian randomization analyses Related to STAR Methods.

**Figure 4:**
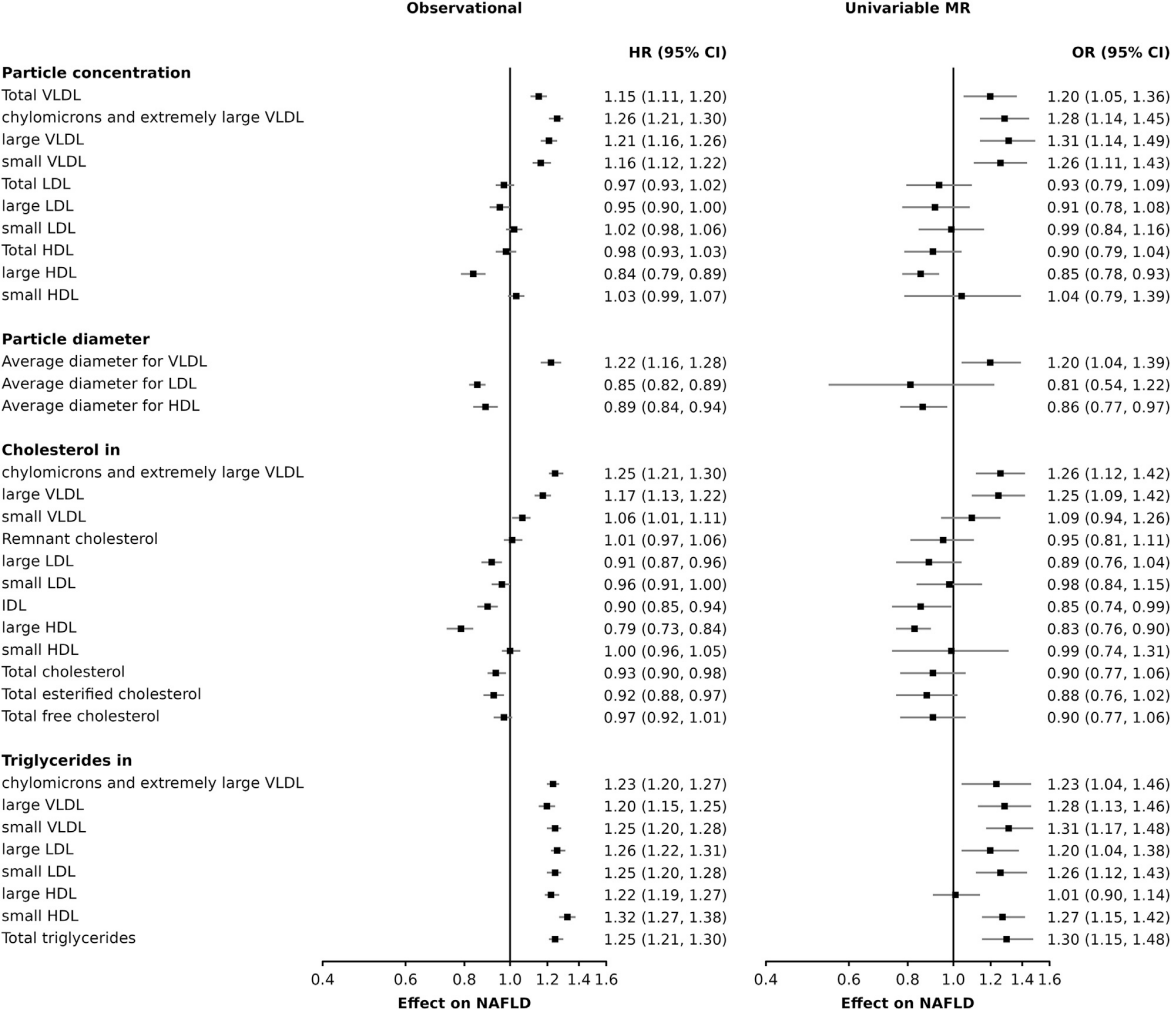
Effect of 1 standard deviation increase in lipoprotein characteristics on non-alcoholic fatty liver disease risk using observational and Mendelian randomization analyses Passed a certain number of exposures (around 40), the forest plot becomes hardly readable. Hence, we had to select a subset of relevant lipoprotein traits. These lipoproteins were selected based on the k-mean clustering analysis which identified two lipoprotein clusters (HDL particle structures and triglyceride levels). We selected relevant traits to enforce this finding. For example, we present triglycerides and triglyceride rich particle concentration as well as LDL and HDL particle concentration. VLDL: very low-density lipoproteins, HDL: high-density lipoproteins; LDL: low-density lipoproteins. For readability, only 33 lipoproteins out of the 132 evaluated are presented. Tables S2 and S5 present all 132 associations. Related to STAR Methods.

We calculated Cox proportional hazards to determine the association of 170 circulating metabolic factors with incident NAFLD. We included age at recruitment, sex, ethnicity, smoking status (ever smoked, never smoked), alcohol consumption, waist circumference as well as medication for blood pressure, diabetes, or cholesterol as covariates. Adjusted hazard ratios (HR) for NAFLD per one standard deviation (SD) increase of circulating metabolites ranged from 0.71 to 1.33. Given the high correlation between each of the exposures evaluated, we adjusted for multiple comparison using principal components. We applied a Bonferroni correction with a number of tests equal to the number of principal components that accounted for a cumulative 90% of the exposure variance (p < 0.05/8 = 0.0063). 112 metabolic factors had evidence for predicting incident NAFLD: 38 metabolites were negatively associated with NAFLD incidence and 74 metabolites were positively associated with NAFLD incidence (Table S1). Figures 2, 3 and 4 present the effect of metabolites, lipids, and lipoprotein concentrations on NAFLD using observational and univariable MR analyses.

### Univariable Mendelian Randomization

We used univariable MR to assess the causal impact of genetically predicted NMR metabolic measures on NAFLD. Table S2 presents the GWAS study details. We selected all robust (p < 5e-8) and independent (linkage disequilibrium [LD] $r^{2}$ < 0.01 in a 1 Mb window) SNPs as genetic instruments for MR analyses. As a sensitivity analysis, we excluded SNPs 500 kb upstream and downstream of the GCKR region (2:27219709-28246554), which is known to exhibit pleiotropy. We used the Steiger’s test to flag and remove genetic instruments that were more strongly linked with the outcome than the exposure. Table S3 presents association statistics for all genetic instruments. All F statistics for the genetic instruments were over 54, indicating excellent strength of genetic instruments (Table S4). We performed IVW-MR and robust MR analyses (MR-PRESSO, weighted median and contamination mixture) for each of the metabolic measures. We used the p value of the intercept test of MR-Egger regression to assess horizontal pleiotropy.

Figure 2 presents the association of 21 circulating metabolites with NAFLD risk in observational and univariable MR analyses. Most metabolites associated with incident NAFLD in observational analyses were not causally associated with NAFLD in MR analyses. Using 14 SNPs ($r^{2}$ = 0.65%; F-statistic = 54), acetate levels were negatively associated with NAFLD (OR 0.46 95% CI = 0.26–0.83, p = 9.3e-03). This association—which was in the opposite direction of the observational point estimate—remained consistent across robust MR analyses (Table S5) and the MR-Egger intercept did not differ significantly from zero (Table S6). However, this association did not pass multiple testing significance. Circulating levels of alanine and albumin were associated with NAFLD using IVW-MR, but results using MVMR, and robust MR were inconclusive.

Figure 3 presents the association of 17 circulating lipids with NAFLD risk in observational and univariable MR analyses. In observational analyses, saturated and monounsaturated fatty acids were positively associated with incident NAFLD, whereas polyunsaturated fatty acids were not associated with incident NAFLD. The ratio of polyunsaturated fatty acids to total fatty acids strongly predicted incident NAFLD (HR 0.71 95% CI = 0.68–0.74, p = 6.1e-54). MR paralleled many of these results, for instance by reporting a strong positive effect of monounsaturated and saturated lipids on NAFLD, but no evidence for an association of polyunsaturated fat and NAFLD. Using a genetic instrument of 56 SNPs ($r^{2}$ = 4.86%; F-statistic = 105), the ratio of polyunsaturated fatty acids to total fatty acids was negatively associated with NAFLD risk (OR = 0.70 95% CI = 0.60–0.81, p = 1.8e-06). The omega-6 to omega-3 ratio, often used as a biomarker for cardiometabolic health, was not associated with incident NAFLD in observational and univariable MR analyses.

Figure 4 presents the association of 33 lipoprotein characteristics and concentration with NAFLD risk in observational and univariable MR analyses. Total cholesterol concentrations were negatively associated with incident NAFLD in observational analyses, but not in MR analyses. By contrast, triglyceride concentrations were positively associated with NAFLD in observational and univariable MR analyses in almost all lipoprotein subfractions. Investigating the association of lipoprotein concentrations and particle diameters, we observed that VLDL particle concentration and diameter were positively associated with NAFLD in observational and univariable MR analyses, LDL particle concentration and diameter were not associated with NAFLD, and large HDL particle concentration and diameter were negatively associated with NAFLD in observational and univariable MR analyses. These results were directionally consistent in robust MR analyses and Egger intercept indicated no significant horizontal pleiotropy.

As several triglyceride-rich lipoprotein characteristics were causally associated with NAFLD, we hypothesized that triglyceride levels may represent the main driver for these associations. Similarly, as several HDL particle content and cholesterol concentrations were causally associated with NAFLD, we hypothesized that HDL-cholesterol may represent the driver for these association. We therefore investigated the association of plasma triglyceride levels and HDL-cholesterol with among participants with these measures available and without NAFLD diagnosis prior to enrollment (458,048 study participants including 8571 incident NAFLD cases). Plasma triglycerides and HDL-cholesterol were associated with NAFLD incidence (HR 1.19 95% CI = 1.17–1.21, p = 1.3e-92 per each mmol/L increase in triglyceride level) and (0.63 95% CI = 0.58–0.69, p = 1.0e-27per each mmol/L increase in HDL-cholesterol level). Tables S7 and S8 presents the dose-dependent effect of plasma triglyceride levels and HDL-cholesterol on incident NAFLD. Participants in the top triglyceride level quintile had an HR of incident NAFLD of 1.96 (95% CI = 1.80–2.13, p = 1.1e-55) compared to participants of the bottom quintile. Participants in the top HDL-cholesterol level quintile had an HR of incident NAFLD of 0.65 (95% CI = 0.59–0.71, p = 3.3e-21) compared to participants in the bottom quintile.

**Figure 5:**
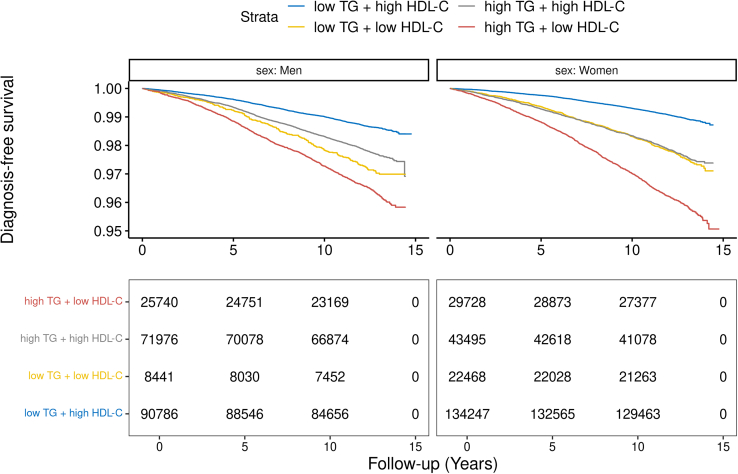
Kaplan-Meier diagnosis-free probability curves for incident non-alcoholic fatty liver disease according to triglyceride and HDL-cholesterol levels There were 225,033 individuals in the low TG + high HDL-C group, 115,471 individuals in the high TG + high HDL-C group, 30,909 individuals in the low TG low HDL-C group, and 55,468 individuals in the high TG + low HDL-C group. Related to STAR Methods.

We also investigated the combined impact of high triglyceride/low HDL-cholesterol metabolic dyslipidemia in the full UK Biobank dataset. We categorized participants based on their lipid levels using clinical cut-off values, as suggested by the National Cholesterol Education Program-Adult Treatment Panel III (NCEP-ATPIII). For TG levels, the cut-off value was 1.7 mmol/L for both men and women and for HDL-C the cut-off value was 1.0 mmol/L for men and 1.3 mmol/L for women. Figure 5 presents the respective time to NAFLD diagnosis Kaplan-Meier curves of these groups. Individuals with metabolic dyslipidemia (high triglycerides and low HDL-cholesterol) were at higher risk of incident NAFLD. In multivariate adjusted Cox regression, participants with metabolic dyslipidemia had an HR of 1.83 (95% CI = 1.72–1.96, p = 7.7e-75) for incident NAFLD compared to participants with low triglycerides and high HDL-cholesterol levels (Table S9). Taken together, results from observational and MR analyses are consistent with a strong and independent causal effect of triglycerides and HDL-cholesterol on NAFLD and suggest that the high triglyceride/low HDL-cholesterol metabolic dyslipidemia may be key risk factors for NAFLD onset.

### Multivariable Mendelian randomization

In a previous MR study, our group identified waist circumference, a crude marker of abdominal adiposity, as a key driver for NAFLD risk.^12^ Waist circumference has been linked, using MR, with circulating lipids and lipoproteins^11^ in the same UK Biobank dataset suggesting that waist circumference could act as a confounding factor in the association between circulating metabolic factors and NAFLD. To remove the effect of waist circumference as a potential confounding factor, we performed multivariable MR. We used as genetic instruments SNPs robustly (pval < 5e-8) and independently (LD *R*^*2*^ < 0.01) associated with each metabolic measure. We included GWAS summary statistics of waist circumference measured in the UK Biobank. We performed MVMR-IVW and several robust MR analyses (MVMR-Lasso, MVMR-Weighted median, MVMR-Egger intercept). The associations for acetate, HDL-cholesterol, fatty acids, and triglycerides remained similar when waist circumference was added in multivariable MR (Figures S1–S3 and Table S10).

**Figure 6:**
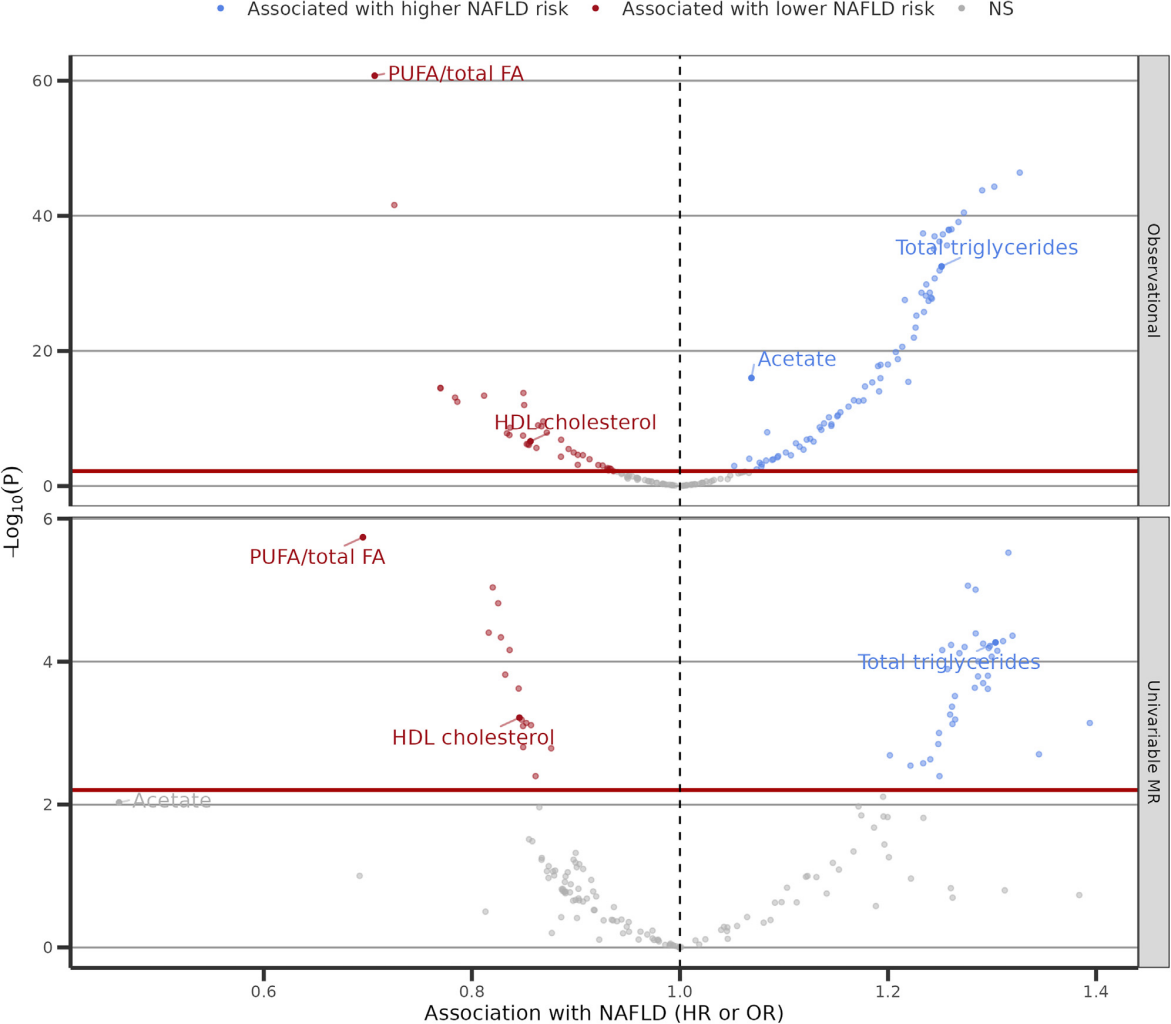
Volcano plot for all metabolic measures evaluated Top panel: prospective observational associations in the UK Biobank (Hazard ratio (HR) presented). Bottom panel: univariable two-sample Mendelian randomization associations (Odds ratio (OR) presented). Related to STAR Methods.

Altogether, we identified 29 metabolic factors that influence NAFLD risk. These associations pass multiple testing correction, are consistent across a range of robust MR analyses, are robust to the inclusion of waist circumference in MVMR analyses and do not display evidence of reverse causality with liver fat accumulation (Methods) (Figures S4–S6). Moreover, none of the genetic variants were flagged by Steiger filtering and the effect sizes remained similar when excluding SNPs in the GCKR gene region (Table S11). Using k-mean clustering on the phenotypic correlation, several of these metabolites are highly correlated and form three clusters (Figure S7). The first cluster encompasses 11 lipoprotein factors related to HDL-cholesterol particles (minimum Pearson r = 0.77), the second encompasses 17 lipoprotein factors related to triglyceride levels (minimum Pearson r = 0.79) and the third is the ratio of PUFA on total FA. All associations are presented in a volcano plot in Figure 6.

## Discussion

This study, the largest to date to investigate incident NAFLD in the general population, examined the association of 21 blood metabolites, 17 lipids, and 132 lipoprotein characteristics with incident and prevalent NAFLD. Using a combination of prospective observational analyses in the UK Biobank and two-sample MR, we identified 29 metabolic factors associated with NAFLD. These associations pass multiple testing correction, are consistent across a range of robust MR analyses, are robust to the inclusion of waist circumference in multivariable MR analyses and do not display evidence of reverse causality with liver fat. These results prioritize triglycerides and triglyceride-rich lipoprotein concentrations, HDL particle structure and PUFA to total FA ratio as key factors that may causally be involved in the onset of NAFLD, which may represent novel therapeutic and dietary strategies to prevent NAFLD. From a clinical standpoint, these results also suggest that the high triglyceride/low HDL-cholesterol metabolic dyslipidemia may be an important risk factor for incident NAFLD.

Previous studies revealed that individuals with NAFLD have higher levels of circulating tyrosine^15^ and branched-chain amino acids (BCAAs: isoleucine, leucine, and valine)^16^ compared to individuals without NAFLD. Similarly, our observational analyses in the UK Biobank support that individuals with high levels of these amino acids are at increased risk of developing NAFLD. However, our MR analyses did not support a causal role for BCAAs or tyrosine on NAFLD. The observational link between BCAAs and NAFLD could arguably be confounded by factors, such as insulin resistance which increase circulating amino acid levels by increasing muscle protein catabolism.^17^ It is worth noting that some preclinical models support a causal role of BCAA in NAFLD development. For instance, overexpression of the phosphatase (PPM1K), which lowers circulating BCAA reduces hepatic steatosis in mice.^18^

Our results support that circulating fatty acids, especially monounsaturated and saturated fatty acids may be positively associated with incident NAFLD and provide some evidence that this association may be causal. The PUFA/total fatty acids ratio was inversely associated with NAFLD risk in observational and MR analyses. Three overfeeding studies reported SFA to increase liver fat more than monounsaturated fat and PUFA. In a study investigating the relative effect of SFA and PUFA, 39 normal-weight individuals were overfed muffins high in saturated fatty acids (palm oil) or n-6 PUFAs (sunflower oil) for seven weeks. Although weight gain was similar between groups, SFA increased liver fat by 40% whereas PUFA did not increase liver fat.^19^ Later in a similar experiment performed in 60 individuals with an elevated body weight, SFA increased liver fat by 50% whereas PUFA did not increase liver fat.^20^ Similarly, in 38 participants considered overweight, overfeeding 1,000 extra kcal/day for three weeks a diet rich in SFA increased liver fat by 55% whereas overfeeding monounsaturated fatty acids only increased liver fat by 15%.^21^ The main limitation from these studies is short duration, leaving the possibility that diet does not have a long-term impact on NAFLD risk. Dietary intervention studies lasting several years are difficult to perform. Our MR results investigating genetically predicted circulating lipids enabled the assessment of lifelong exposure to various blood lipid concentrations. Results of these analyses support those of dietary interventions studies and confirm that diets rich in PUFA and low in SFA may contribute to NAFLD prevention/treatment.

Our results support that high levels of triglycerides may be linked with NAFLD incidence (with UK Biobank participants in the top triglyceride quintile being at more than 2-fold higher NAFLD risk compared to those of the bottom quintile) but also that this relationship may be causal. Triglycerides lowering may offer promise for NAFLD prevention. For example, the thiazolidinedione pioglitazone decreases steatosis by binding and activating peroxisome proliferator-activated receptor gamma in adipocytes to promote adipogenesis and fatty acid uptake in peripheral but not visceral fat.^22^ In 55 randomly assigned patients with NASH, pioglitazone plus a hypocaloric diet, decreased triglyceride levels and hepatic fat content by 54% compared to hypocaloric diet plus placebo.^23^ Similarly, in 101 patients with NASH hypocaloric diet plus pioglitazone, as compared with hypocaloric diet plus placebo resolved NASH in 51% of pioglitazone-treated patients vs. 19% of placebo-treated patients. Hepatic triglyceride content and circulating triglyceride levels also both decreased.^24^ Another triglyceride-lowering agent, fenofibrate, reduces circulating triglycerides primarily by increasing B-oxidation and triglyceride-rich lipoprotein catabolism^25^ (whereas pioglitazone increase adipose tissue storage capacity). In several small clinical studies, fenofibrate significantly lowered circulating very low-density lipoprotein-TG (VLDL-TG) concentrations by as much as 48.9%, but the hepatic lipid accumulation remained unchanged.^26^^,^^27^^,^^28^^,^^29^ Recently, the PROMINENT trial demonstrated that pemafibrate treatment reduced triglyceride levels by 26% and reduced investigator-reported 3.4 years incident NAFLD by 22% when compared to placebo in 10,497 patients with type 2 diabetes.^30^ These studies, combined with the results of our study suggest that the mechanism of triglyceride-reducing drugs may be important for the prevention and/or treatment of NAFLD.

Although CAD and NAFLD are both associated with dyslipidemia, CAD lipoprotein/lipids causal factors may differ from NAFLD’s. The levels of triglycerides and cholesterol are intertwined because of their shared metabolism. Alterations in both LDL and triglycerides are associated with CAD incidence; although drugs that reduce LDL have been successful in preventing CAD, drugs that reduce triglyceride levels have not shown consistent effects.^31^ Multivariable MR analyses reached similar conclusions, prioritizing apolipoprotein B, a key determinant of atherogenic lipoprotein particles, as the main causal factor for CAD.^9^^,^^32^ Our MR analysis revealed no causal effect of LDL cholesterol on NAFLD risk, but a large effect of triglycerides on NAFLD risk. Hence, among lipids, LDL-C/APOB is the main causal factor for CAD, but triglycerides and HDL particle composition appear to be important causal factors for NAFLD.

The main strength of this paper is the combination of observational and MR analyses and the use of the largest study sample to date to investigate the relationship between blood metabolites, lipids, and lipoproteins with incident (rather than prevalent) NAFLD. At the present time, MR methods cannot analyze time-to-event data (although this is an area of intensive research). Hence, the two methods are complementary. MR enables the study of lifelong genetic effect of metabolic factors on NAFLD in a large European sample, whereas prospective observational analyses evaluate predictive value of metabolic factors on incident NAFLD.

In conclusion, we have screened 170 metabolic factors for their association with incident NAFLD using a combination of observational and MR analyses. This analysis revealed that many of these metabolites such as BCAAs are associated with incident NAFLD but that they may not be causal risk factors for NAFLD. On the other hand, several plasma lipids and lipoproteins provided concordant effects on NAFLD in observational and MR analyses. Our results suggest that therapies aimed at reducing circulating triglyceride-rich lipoprotein concentrations or increasing HDL particles as well as interventions targeting dietary fat quality, for instance by increasing polyunsaturated over other fats, could prevent the onset of NAFLD.

### Limitations of the study

Our findings should be considered within the study limits. First, he NAFLD GWAS included ∼8000 cases and ∼700,000 controls and there were 11,308 cases and 491,103 controls in the entire UK Biobank sample, but the population prevalence of NAFLD in the UK has been estimated to be around 25%. Furthermore, we used liver fat percentage measured by liver magnetic resonance imaging in the UK Biobank^33^^,^^34^ to derive a more accurate NAFLD prevalence estimate in the UK Biobank. The proportion of individuals with a liver fat percentage > 5% was 10967/40533 or 27% of the sample. Hence, it is highly probable that some controls may have been misclassified. While it is important to acknowledge this limitation, we believe that such misclassification could bias our results toward the null and underestimate the strength of the reported associations. Second, our MR analysis was restricted to participants of European ancestry only, because of the scarce availability of genome-wide summary statistics on non-European individuals. While this might restrict the generalizability of our results to this group, our observational analyses, performed without excluding non-white individuals, yielded similar conclusions. Finally, the omega-6 to omega-3 ratio was not associated with incident NAFLD. We did not have access to EPA/DHA ratio and hepatic measures, which may explain the null association.

## STAR★Methods

### Key resources table

**Table:** 

REAGENT or RESOURCE	SOURCE	IDENTIFIER
**Deposited Data**		

NMR_Metabolites	Borges, Maria Carolina, Philip C. Haycock, Jie Zheng, Gibran Hemani, Michael V. Holmes, George Davey Smith, Aroon D. Hingorani, and Deborah A. Lawlor. 2022. ‘Role of Circulating Polyunsaturated Fatty Acids on Cardiovascular Diseases Risk: Analysis Using Mendelian Randomization and Fatty Acid Genetic Association Data from over 114,000 UK Biobank Participants’. *BMC Medicine* 20 (1): 210. https://doi.org/10.1186/s12916-022-02399-w.	https://gwas.mrcieu.ac.uk/files/met-d-PUFA_pct/met-d-PUFA_pct.vcf.gz
Waist circumference	Hemani, Gibran, Jie Zheng, Benjamin Elsworth, Kaitlin H Wade, Valeriia Haberland, Denis Baird, Charles Laurin, et al. 2018. ‘The MR-Base Platform Supports Systematic Causal Inference across the Human Phenome’. *ELife* 7 (May): e34408. https://doi.org/10.7554/eLife.34408	https://gwas.mrcieu.ac.uk/ukb-b-9405/ukb-b-9405.vcf.gz
NAFLD	Ghodsian, Nooshin, Erik Abner, Connor A. Emdin, Émilie Gobeil, Nele Taba, Mary E. Haas, Nicolas Perrot, et al. 2021. ‘Electronic Health Record-Based Genome-Wide Meta-Analysis Provides Insights on the Genetic Architecture of Non-Alcoholic Fatty Liver Disease’. *Cell Reports*. *Medicine* 2 (11): 100437. https://doi.org/10.1016/j.xcrm.2021.100437.	https://www.ebi.ac.uk/gwas/publications/34841290
Liver_Fat	Liu, Yi, Nicolas Basty, Brandon Whitcher, Jimmy D Bell, Elena P Sorokin, Nick van Bruggen, E Louise Thomas, and Madeleine Cule. 2021. ‘Genetic Architecture of 11 Organ Traits Derived from Abdominal MRI Using Deep Learning’. Edited by Edward D Janus, Matthias Barton, and Constantinos Parisinos. *ELife* 10 (June): e65554. https://doi.org/10.7554/eLife.65554	http://ftp.ebi.ac.uk/pub/databases/gwas/summary_statistics/GCST90016001-GCST90017000/GCST90016676/.

**Software and Algorithms**		

TwoSampleMR V.0.5.6 (R package)	Hemani, G., Zheng, J., Elsworth, B., Wade, K.H., Haberland, V., Baird, D., Laurin, C., Burgess, S., Bowden, J., Langdon, R., et al. (2018). The MR-Base platform supports systematic causal inference across the human phenome. eLife *7*, e34408. 10.7554/eLife.34408.	https://github.com/MRCIEU/TwoSampleMR
data.table V.1.14.0 (R package)	Dowle, M. and Srinivasan, A., 2019. data. table: Extension of ‘data. frame’. R package version 1.12. 8.*Manual*.	https://github.com/Rdatatable/data.table
gwasglue V.0.0.0.9000	Elsworth, B., Lyon, M., Alexander, T., Liu, Y., Matthews, P., Hallett, J., Bates, P., Palmer, T., Haberland, V., Smith, G.D., et al. (2020). The MRC IEU OpenGWAS data infrastructure. 2020.08.10.244293. 10.1101/2020.08.10.244293.	https://rdrr.io/github/MRCIEU/gwasglue/
gwasvcf V.0.1.0	Elsworth, B., Lyon, M., Alexander, T., Liu, Y., Matthews, P., Hallett, J., Bates, P., Palmer, T., Haberland, V., Smith, G.D., et al. (2020). The MRC IEU OpenGWAS data infrastructure. 2020.08.10.244293. 10.1101/2020.08.10.244293.	https://github.com/MRCIEU/gwasvcf
MendelianRandomization V 0.7.0	Yavorska, O.O., and Burgess, S. (2017). MendelianRandomization: an R package for performing Mendelian randomization analyses using summarized data. Int J Epidemiol *46*, 1734–1739. 10.1093/ije/dyx034.	https://cran.r-project.org/web/packages/MendelianRandomization/index.html

### Resource availability

#### Lead contact

Further information and request for resources should be directed to and will be fulfilled by the lead contact, Benoit J. Arsenault (benoit.arsenault@criucpq.ulaval.ca).

#### Materials availability

This study did not generate new material or reagent.

### Experimental model and study participant details

#### Study participants

The UK Biobank is a population-based cohort of approximately 500,000 participants aged 40 to 69 years recruited during 2006–2010 from several centers across the UK. Data access permission for this study was granted under UKB application 25205. NMR metabolic biomarkers have been quantified in approximately one third of randomly selected participants in the UK Biobank.^14^ Metabolites from non-fasted plasma samples were measured using high-throughput nuclear magnetic resonance (NMR) (Nightingale Health Ltd; biomarker quantification version 2020). A total of 121,074 participants (46% men, 94% White) were retained for analyses after removing duplicates and observations not passing quality control (QC) (i.e., sample QC flag “low protein,” biomarker QC flag “technical error” or samples with insufficient material). NMR platform quantifies 168 metabolic measures and 81 derived ratios. We included all metabolic measures and two ratios: the ratio of polyunsaturated fatty acids over all fatty acids and the ratio of Omega 6 fatty acids over Omega 3 fatty acids. The NMR platform utilization has been previously described.^14^ We used the *ukbnmr* package for quality control and removal of technical variation of the metabolites.^35^

### Method details

#### Observational analyses

We used first occurrences dates for liver disease ("K74," "K75," "K760," ICD-10) provided by UKB until October 1, 2021. These data were retrieved from death registries, primary care information (available for ∼45% of UKB participants), hospital admission medical reports and self-report data. We then used Hospital Episode Statistics data to refine our variable to ICD10 code at three levels by excluding participants with hospital inpatient exclusion code (all K.74 code except K74.0 and K74.2 (hepatic fibrosis), all K75 code except K75.8 (NASH), all K76 code except K76.0 (NAFLD) and ICD10: K76.9 (other specified diseases of the liver)). This procedure allows the exclusion of participants with liver disease that were not related to NAFLD (alcoholic liver disease, viral hepatitis, alpha-1 anti trypsin deficiency, etc.). NAFLD Cox regression results were obtained from models adjusted for variables at recruitment including age, sex, smoking status (never/ever smoked), alcohol consumption, Townsend’s deprivation index, ethnicity, waist circumference, and metabolic syndrome medication dichotomized (do you presently take any of the following medications: cholesterol-lowering medication, blood pressure medication or insulin). Results are presented as estimated hazard ratios (HR) for incident NAFLD diagnosis per SD increase of circulating metabolites, with 95% confidence intervals. We also plotted Kaplan-Meier survival curves for triglyceride and HDL-cholesterol quintiles as well as for the combination of the two risk factors.

#### GWAS summary statistics for MR analysis

Table S1 presents the study characteristics of the GWAS used in this MR analysis. NALFD: We previously performed a GWAS meta-analysis for electronic health record of NAFLD (8434 cases and 770,180 controls) of European ancestry.^36^ We defined NAFLD cases with medical diagnosis ICD10 code (K74.0 and K74.2 (hepatic fibrosis), K75.8 (NASH), K76.0 (NAFLD) and ICD10: K76.9 (other specified diseases of the liver). Logistic regression was performed at each SNP with adjustment for age, sex, genotyping site and the first three ancestries-based principal components. We then meta-analyzed four cohorts (The eMERGE network, the UK Biobank, the Estonian Biobank and FinnGen) using *METAL*.^37^ Liver Fat: GWAS summary statistics for liver fat measured using magnetic resonance imaging were obtained from a GWAS of 32,860 white British participants from the UK Biobank.^38^ Magnetic resonance scans were annotated by trained radiologists following a standard procedure. Deep learning algorithms were then applied to estimate liver fat. Liver fat was inverse-normal transformed and adjusted for genetic sex, age, age squared, the first 10 principal components of genetic ancestry, scaled scan date, scaled scan time, and study center as fixed effects and genetic relatedness as a random effects term. Using BOLT-LMM, the residuals were then regressed on gene carrier status. Waist circumference: GWAS summary statistics for waist circumference were obtained from the UK Biobank from 462,166 Europeans. GWAS was conducted using linear mixed model (LMM) association method as implemented in BOLT-LMM (v2.3) correcting genotype array, sex and the ten first genetic principal components. The resulting residuals were inverse-normal transformed prior to GWAS. Circulating metabolic measures: GWAS summary statistics for 170 circulating metabolites were obtained from 114,999 UK Biobank participants of European ancestry.^39^ GWAS was conducted using LMM association method as implemented in BOLT-LMM (v2.3) correcting genotype array, sex and the ten first genetic principal components. All measures were standardized and normalized prior to analyses using rank-based inverse-normal transformation. These summary statistics were accessed through the IEU Open GWAS Project.

### Quantification and statistical analysis

#### Univariable MR analyses

For univariable MR analyses, we included as genetic instruments all genome-wide significant SNPs (p value <5e-8). We then ensured their independence by clumping using 1 Mb window with a LD $r^{2}$ <0.01 using the European 1000-genome LD reference panel. We excluded genetic regions that are known to be widely pleiotropic with a primary effect on the liver to take MR assumptions into consideration. For this reason, we removed the GCKR region as a sensitivity analysis. GCKR is primarily expressed in the liver, where it regulates lipogenesis, glycogenesis and B-oxidation. A non-synonymous genetic variant in the GCKR region is known to influence the levels of a wide range of metabolites.^40^ Table S2 contains relevant association statistics for genetics instruments of each exposure. We harmonized the exposure and outcome datasets by aligning the effect sizes of different studies on the same effect allele. All GWAS summary statistics were reported on the forward strand. When a particular SNP was not present in the outcome datasets, we used a proxy SNP ($r^{2}$ > 0.8) obtained using LD matrix of European samples from the 1000 Genomes Project.

We performed the inverse variance weighted (IVW) method with multiplicative random effects with a standard error correction for under dispersion as primary MR analysis. MR must respect three core assumptions (relevance, exchangeability, and exclusion restriction) to assert correct causal inference. We evaluated the relevance assumption by assessing instrument strength using the F-statistic,^41^ and the variance explained using the $r^{2}$^42^ (Table S6). The exchangeability and the exclusion restriction assumption are unverifiable but can be questioned when heterogeneity in the estimate is present. We quantified heterogeneity in the exposure outcome association using Cochran’s Q. We also perform four different robust MR analyses (the MR Egger,^10^ the contamination mixture,^43^ the weighted median, and the MR-PRESSO^44^) to explore whether the results differed from the primary MR results. These robust methods, their assumptions and their statistical properties have been extensively reviewed elsewhere.^45^ Given the high correlation between each of the exposures evaluated, we adjusted for multiple comparison using principal components. We applied a Bonferroni correction with a number of tests equal to the number of principal components that accounted for a cumulative 90% of the exposure variances. In our dataset, the first eight principal component accounted for 90% of the exposure’s variance. Therefore, we applied a Bonferroni correction for 8 tests throughout the manuscript. We considered as potential causal association the MR results with significant primary MR analysis after adjustment for multiple testing, with a majority of robust MR analyses significant at p < 0.05, with all estimates directionally consistent across methods, with Egger intercept p < 0.05, with robustness to the inclusion of waist circumference in MVMR, and with no evidence for reverse causality with liver fat.

We evaluated reverse causality by performing Steiger filtering and reverse MR. The Steiger test provides a p value under the null hypothesis that the difference in variance explained is null.^46^ We systematically removed the variants tagged by the Steiger test. As another test of reverse causality, we performed reverse MR. It is not recommended to perform MR with a binary factor as exposure.^47^ The binary factor NAFLD, which is diagnosed when liver fat percentage is above 5%, is akin to a dichotomization of the underlying continuous factor “liver fat”. We therefore estimated the causal effect of the continuous variable “liver fat”, and not NAFLD, in reverse MR.

#### Multivariable MR analyses

For multivariable MR, we included as genetic instruments the SNPs that were previously selected in univariable MR for the exposure (i.e., metabolites, lipid concentrations and lipoprotein characteristics). We used the same set of SNPs for the covariate (i.e., waist circumference) and harmonized the effect allele with the exposure SNPs. When an SNP was not present, either in the exposure or in the outcome dataset, we used a proxy SNP ($r^{2}$ > 0.8) instead. As primary multivariable MR analysis, we performed the IVW method. Like univariable MR, Multivariable MR must include valid instruments that respect the three core assumptions. In contrast to univariable MR, multivariable MR allows genetic variants to be associated with multiple risk factors (pleiotropy) given that these risk factors are included in the analysis. We evaluated the relevance assumption by quantifying instrument strength using the conditional F-statistics^48^ in the *MVMR* V.0.2.0 package.^48^ We quantified heterogeneity in the exposure outcome association correcting for covariates using Cochran’s Q.^13^ We also performed two different robust MR analyses: the multivariable median method and the multivariable MR-Lasso method.^49^ Like robust univariable MR analyses, consistent estimates across different methods give confidence in the robustness of the causal findings. We considered as potential causal association the MR results that were supported by primary MVMR analysis and robust MVMR analyses (MVMR-Weighted median method p < 0.05, MVMR-Egger intercept p > 0.05, MVMR-Lasso p < 0.05) and all estimates directionally consistent.

#### Institutional review board approval

The UK Biobank received ethical approval from the Research Ethics Committee (REC reference for the UK Biobank is 11/NW/0382). All GWAS summary statistics were publicly available and accessible through URL. For all included genetic association studies, all participants provided informed consent and study protocols were approved by their respective local ethical committee.

## Data Availability

• All genome-wide summary statistics used in this study are in the public domain. • All original code has been deposited on GitHub and is publicly available as of the date of publication at https://github.com/LaboArsenault/NMR_NAFLD.git • Any additional information required to reanalyze the data reported in this paper is available from the lead contact upon request.

## Supplemental information

Document S1. Figures S1–S7

Table S1. Cox Hazard ratio results for all 170 metabolic factors on NAFLD adjusted for age enrollment, ethnicity, sex, medicine, smoking, alcohol, Townsend deprivation index, waist circumference. Related to STAR methods

Table S2. Description of the datasets used for Two-Sample Mendelian randomization, related to STAR methods

Table S3. Instruments and relevant statistics for each exposure and each SNPs, related to STAR methods

Table S4. Instrument strength (F-statistics) and heterogeneity statistics (Cochran’s Q) for univariable MR, related to STAR methods

Table S5. Univariable Mendelian Randomization results using different pleiotropy robust MR analyses, related to STAR methods

Table S6. Univariable Mendelian Randomization Egger’s intercept, related to STAR methods

Table S7. Event rates and hazard ratios for non-alcoholic fatty liver disease in UK Biobank participants for the entire cohort, as well as in men and women separately depending on triglyceride quintile, related to STAR methods

Table S8. Event rates and hazard ratios for non-alcoholic fatty liver disease in UK Biobank participants for the entire cohort, as well as in men and women separately, depending on HDL quintile, related to STAR methods

Table S9. Event rates and hazard ratios for non-alcoholic fatty liver disease in UK Biobank participants for the entire cohort are presented, as well as in men and women separately, depending on HDL cholesterol and triglyceride levels, related to STAR methods

Table S10. Multivariable Mendelian randomization results using different pleiotropy robust MVMR analyses, related to STAR methods

Table S11. Inverse variance weighted MR result for all metabolic measures with removing GCKR region, related to STAR methods

## References

[1] Kanwal F., Kramer J.R., Mapakshi S., Natarajan Y., Chayanupatkul M., Richardson P.A., Li L., Desiderio R., Thrift A.P., Asch S.M. (2018). Risk of Hepatocellular Cancer in Patients With Non-Alcoholic Fatty Liver Disease. Gastroenterology.

[2] Araújo A.R., Rosso N., Bedogni G., Tiribelli C., Bellentani S. (2018). Global epidemiology of non-alcoholic fatty liver disease/non-alcoholic steatohepatitis: What we need in the future. Liver Int..

[3] Lazarus J.V., Mark H.E., Anstee Q.M., Arab J.P., Batterham R.L., Castera L., Cortez-Pinto H., Crespo J., Cusi K., Dirac M.A. (2022). Advancing the global public health agenda for NAFLD: a consensus statement. Nat. Rev. Gastroenterol. Hepatol..

[4] Godoy-Matos A.F., Silva Júnior W.S., Valerio C.M. (2020). NAFLD as a continuum: from obesity to metabolic syndrome and diabetes. Diabetol. Metab. Syndr..

[5] Masoodi M., Gastaldelli A., Hyötyläinen T., Arretxe E., Alonso C., Gaggini M., Brosnan J., Anstee Q.M., Millet O., Ortiz P. (2021). Metabolomics and lipidomics in NAFLD: biomarkers and non-invasive diagnostic tests. Nat. Rev. Gastroenterol. Hepatol..

[6] Gaggini M., Carli F., Rosso C., Buzzigoli E., Marietti M., Della Latta V., Ciociaro D., Abate M.L., Gambino R., Cassader M. (2018). Altered amino acid concentrations in NAFLD: Impact of obesity and insulin resistance. Hepatology.

[7] Männistö V.T., Simonen M., Soininen P., Tiainen M., Kangas A.J., Kaminska D., Venesmaa S., Käkelä P., Kärjä V., Gylling H. (2014). Lipoprotein subclass metabolism in nonalcoholic steatohepatitis. J. Lipid Res..

[8] Pang Y., Kartsonaki C., Lv J., Millwood I.Y., Fairhurst-Hunter Z., Turnbull I., Bragg F., Hill M.R., Yu C., Guo Y. (2022). Adiposity, metabolomic biomarkers, and risk of nonalcoholic fatty liver disease: a case-cohort study. Am. J. Clin. Nutr..

[9] Zuber V., Gill D., Ala-Korpela M., Langenberg C., Butterworth A., Bottolo L., Burgess S. (2021). High-throughput multivariable Mendelian randomization analysis prioritizes apolipoprotein B as key lipid risk factor for coronary artery disease. Int. J. Epidemiol..

[10] Bowden J., Davey Smith G., Burgess S. (2015). Mendelian randomization with invalid instruments: effect estimation and bias detection through Egger regression. Int. J. Epidemiol..

[11] Bell J.A., Richardson T.G., Wang Q., Sanderson E., Palmer T., Walker V., O’Keeffe L.M., Timpson N.J., Cichonska A., Julkunen H. (2022). Effects of general and central adiposity on circulating lipoprotein, lipid, and metabolite levels in UK Biobank: A multivariable Mendelian randomization study. Lancet Reg. Health. Eur..

[12] Gagnon E., Pelletier W., Gobeil É., Bourgault J., Manikpurage H.D., Maltais-Payette I., Abner E., Taba N., Esko T., Mitchell P.L. (2022). Mendelian randomization prioritizes abdominal adiposity as an independent causal factor for liver fat accumulation and cardiometabolic diseases. Commun. Med..

[13] Burgess S., Thompson S.G. (2015). Multivariable Mendelian Randomization: The Use of Pleiotropic Genetic Variants to Estimate Causal Effects. Am. J. Epidemiol..

[14] Julkunen H., Cichońska A., Slagboom P.E., Würtz P., Nightingale Health UK Biobank Initiative (2021). Metabolic biomarker profiling for identification of susceptibility to severe pneumonia and COVID-19 in the general population. Elife.

[15] Gobeil É., Maltais-Payette I., Taba N., Brière F., Ghodsian N., Abner E., Bourgault J., Gagnon E., Manikpurage H.D., Couture C. (2022). Mendelian Randomization Analysis Identifies Blood Tyrosine Levels as a Biomarker of Non-Alcoholic Fatty Liver Disease. Metabolites.

[16] Chashmniam S., Ghafourpour M., Rezaei Farimani A., Gholami A., Nobakht Motlagh Ghoochani B.F. (2019). Metabolomic Biomarkers in the Diagnosis of Non-Alcoholic Fatty Liver Disease. Hepat. Mon..

[17] Lynch C.J., Adams S.H. (2014). Branched-chain amino acids in metabolic signalling and insulin resistance. Nat. Rev. Endocrinol..

[18] White P.J., McGarrah R.W., Grimsrud P.A., Tso S.-C., Yang W.-H., Haldeman J.M., Grenier-Larouche T., An J., Lapworth A.L., Astapova I. (2018). The BCKDH Kinase and Phosphatase Integrate BCAA and Lipid Metabolism via Regulation of ATP-Citrate Lyase. Cell Metab..

[19] Rosqvist F., Iggman D., Kullberg J., Cedernaes J., Johansson H.-E., Larsson A., Johansson L., Ahlström H., Arner P., Dahlman I., Risérus U. (2014). Overfeeding polyunsaturated and saturated fat causes distinct effects on liver and visceral fat accumulation in humans. Diabetes.

[20] Rosqvist F., Kullberg J., Ståhlman M., Cedernaes J., Heurling K., Johansson H.-E., Iggman D., Wilking H., Larsson A., Eriksson O. (2019). Overeating Saturated Fat Promotes Fatty Liver and Ceramides Compared With Polyunsaturated Fat: A Randomized Trial. J. Clin. Endocrinol. Metab..

[21] Luukkonen P.K., Sädevirta S., Zhou Y., Kayser B., Ali A., Ahonen L., Lallukka S., Pelloux V., Gaggini M., Jian C. (2018). Saturated Fat Is More Metabolically Harmful for the Human Liver Than Unsaturated Fat or Simple Sugars. Diabetes Care.

[22] Greenfield J.R., Chisholm D.J. (2004).

[23] Belfort R., Harrison S.A., Brown K., Darland C., Finch J., Hardies J., Balas B., Gastaldelli A., Tio F., Pulcini J. (2006). A Placebo-Controlled Trial of Pioglitazone in Subjects with Nonalcoholic Steatohepatitis. N. Engl. J. Med..

[24] Cusi K., Orsak B., Bril F., Lomonaco R., Hecht J., Ortiz-Lopez C., Tio F., Hardies J., Darland C., Musi N. (2016). Long-Term Pioglitazone Treatment for Patients With Nonalcoholic Steatohepatitis and Prediabetes or Type 2 Diabetes Mellitus. Ann. Intern. Med..

[25] Shepherd J., Packard C.J., Stewart J.M., Atmeh R.F., Clark R.S., Boag D.E., Carr K., Lorimer A.R., Ballantyne D., Morgan H.G. (1984). Apolipoprotein A and B (Sf 100-400) metabolism during bezafibrate therapy in hypertriglyceridemic subjects. J. Clin. Invest..

[26] Fernández-Miranda C., Pérez-Carreras M., Colina F., López-Alonso G., Vargas C., Solís-Herruzo J.A. (2008). A pilot trial of fenofibrate for the treatment of non-alcoholic fatty liver disease. Dig. Liver Dis..

[27] El-Haggar S.M., Mostafa T.M. (2015). Comparative clinical study between the effect of fenofibrate alone and its combination with pentoxifylline on biochemical parameters and liver stiffness in patients with non-alcoholic fatty liver disease. Hepatol. Int..

[28] Fabbrini E., Mohammed B.S., Korenblat K.M., Magkos F., McCrea J., Patterson B.W., Klein S. (2010). Effect of fenofibrate and niacin on intrahepatic triglyceride content, very low-density lipoprotein kinetics, and insulin action in obese subjects with nonalcoholic fatty liver disease. J. Clin. Endocrinol. Metab..

[29] Oscarsson J., Önnerhag K., Risérus U., Sundén M., Johansson L., Jansson P.-A., Moris L., Nilsson P.M., Eriksson J.W., Lind L. (2018). Effects of free omega-3 carboxylic acids and fenofibrate on liver fat content in patients with hypertriglyceridemia and non-alcoholic fatty liver disease: A double-blind, randomized, placebo-controlled study. J. Clin. Lipidol..

[30] Das Pradhan A., Glynn R.J., Fruchart J.-C., MacFadyen J.G., Zaharris E.S., Everett B.M., Campbell S.E., Oshima R., Amarenco P., Blom D.J. (2022). Triglyceride Lowering with Pemafibrate to Reduce Cardiovascular Risk. N. Engl. J. Med. Overseas. Ed..

[31] Jakob T., Nordmann A.J., Schandelmaier S., Ferreira-González I., Briel M. (2016). Fibrates for primary prevention of cardiovascular disease events. Cochrane Database Syst. Rev..

[32] Richardson T.G., Sanderson E., Palmer T.M., Ala-Korpela M., Ference B.A., Davey Smith G., Holmes M.V. (2020). Evaluating the relationship between circulating lipoprotein lipids and apolipoproteins with risk of coronary heart disease: A multivariable Mendelian randomisation analysis. PLoS Med..

[33] Parisinos C.A., Wilman H.R., Thomas E.L., Kelly M., Nicholls R.C., McGonigle J., Neubauer S., Hingorani A.D., Patel R.S., Hemingway H. (2020). Genome-wide and Mendelian randomisation studies of liver MRI yield insights into the pathogenesis of steatohepatitis. J. Hepatol..

[34] Wilman H.R., Kelly M., Garratt S., Matthews P.M., Milanesi M., Herlihy A., Gyngell M., Neubauer S., Bell J.D., Banerjee R., Thomas E.L. (2017). Characterisation of liver fat in the UK Biobank cohort. PLoS One.

[35] Ritchie S.C., Surendran P., Karthikeyan S., Lambert S.A., Bolton T., Pennells L., Danesh J., Di Angelantonio E., Butterworth A.S., Inouye M. (2023). Quality control and removal of technical variation of NMR metabolic biomarker data in ∼120,000 UK Biobank participants. Sci. Data.

[36] Ghodsian N., Abner E., Emdin C.A., Gobeil É., Taba N., Haas M.E., Perrot N., Manikpurage H.D., Gagnon É., Bourgault J. (2021). Electronic health record-based genome-wide meta-analysis provides insights on the genetic architecture of non-alcoholic fatty liver disease. Cell Rep. Med..

[37] Willer C.J., Li Y., Abecasis G.R. (2010). METAL: fast and efficient meta-analysis of genomewide association scans. Bioinformatics.

[38] Liu Y., Basty N., Whitcher B., Bell J.D., Sorokin E.P., van Bruggen N., Thomas E.L., Cule M. (2021). Genetic architecture of 11 organ traits derived from abdominal MRI using deep learning. Elife.

[39] Borges M.C., Haycock P.C., Zheng J., Hemani G., Holmes M.V., Davey Smith G., Hingorani A.D., Lawlor D.A. (2022). Role of circulating polyunsaturated fatty acids on cardiovascular diseases risk: analysis using Mendelian randomization and fatty acid genetic association data from over 114,000 UK Biobank participants. BMC Med..

[40] Lotta L.A., Pietzner M., Stewart I.D., Wittemans L.B.L., Li C., Bonelli R., Raffler J., Biggs E.K., Oliver-Williams C., Auyeung V.P.W. (2021). A cross-platform approach identifies genetic regulators of human metabolism and health. Nat. Genet..

[41] Burgess S., Thompson S.G., CRP CHD Genetics Collaboration (2011). Avoiding bias from weak instruments in Mendelian randomization studies. Int. J. Epidemiol..

[42] Pierce B.L., Ahsan H., VanderWeele T.J. (2011). Power and instrument strength requirements for Mendelian randomization studies using multiple genetic variants. Int. J. Epidemiol..

[43] Burgess S., Foley C.N., Allara E., Staley J.R., Howson J.M.M. (2020). A robust and efficient method for Mendelian randomization with hundreds of genetic variants. Nat. Commun..

[44] Verbanck M., Chen C.-Y., Neale B., Do R. (2018). Detection of widespread horizontal pleiotropy in causal relationships inferred from Mendelian randomization between complex traits and diseases. Nat. Genet..

[45] Slob E.A.W., Burgess S. (2020). A comparison of robust Mendelian randomization methods using summary data. Genet. Epidemiol..

[46] Hemani G., Tilling K., Davey Smith G. (2017). Orienting the causal relationship between imprecisely measured traits using GWAS summary data. PLoS Genet..

[47] Burgess S., Labrecque J.A. (2018). Mendelian randomization with a binary exposure variable: interpretation and presentation of causal estimates. Eur. J. Epidemiol..

[48] Sanderson E., Spiller W., Bowden J. (2021). Testing and correcting for weak and pleiotropic instruments in two-sample multivariable Mendelian randomization. Stat. Med..

[49] Grant A.J., Burgess S. (2021). Pleiotropy robust methods for multivariable Mendelian randomization. Stat. Med..

