# Supplementary material for: Large-scale metabolomic profiling and incident non-alcoholic fatty liver disease
Source: iScience. 2023 Jun 14;26(7):107127. doi: 10.1016/j.isci.2023.107127 (PMC10339047; doi:10.1016/j.isci.2023.107127)
Supplement: Document S1. Figures S1–S7 [file mmc1.pdf]

iScience, Volume *26*

Supplemental information

Large-scale metabolomic profiling and incident non-alcoholic fatty liver disease

Eloi Gagnon, Hasanga D. Manikpurage, Patricia L. Mitchell, Arnaud Girard, Émilie Gobeil, Jérôme Bourgault, Frédéric Bégin, André Marette, Sébastien Thériault, and Benoit J. Arsenault

Supplementary Material

Supplementary Figures

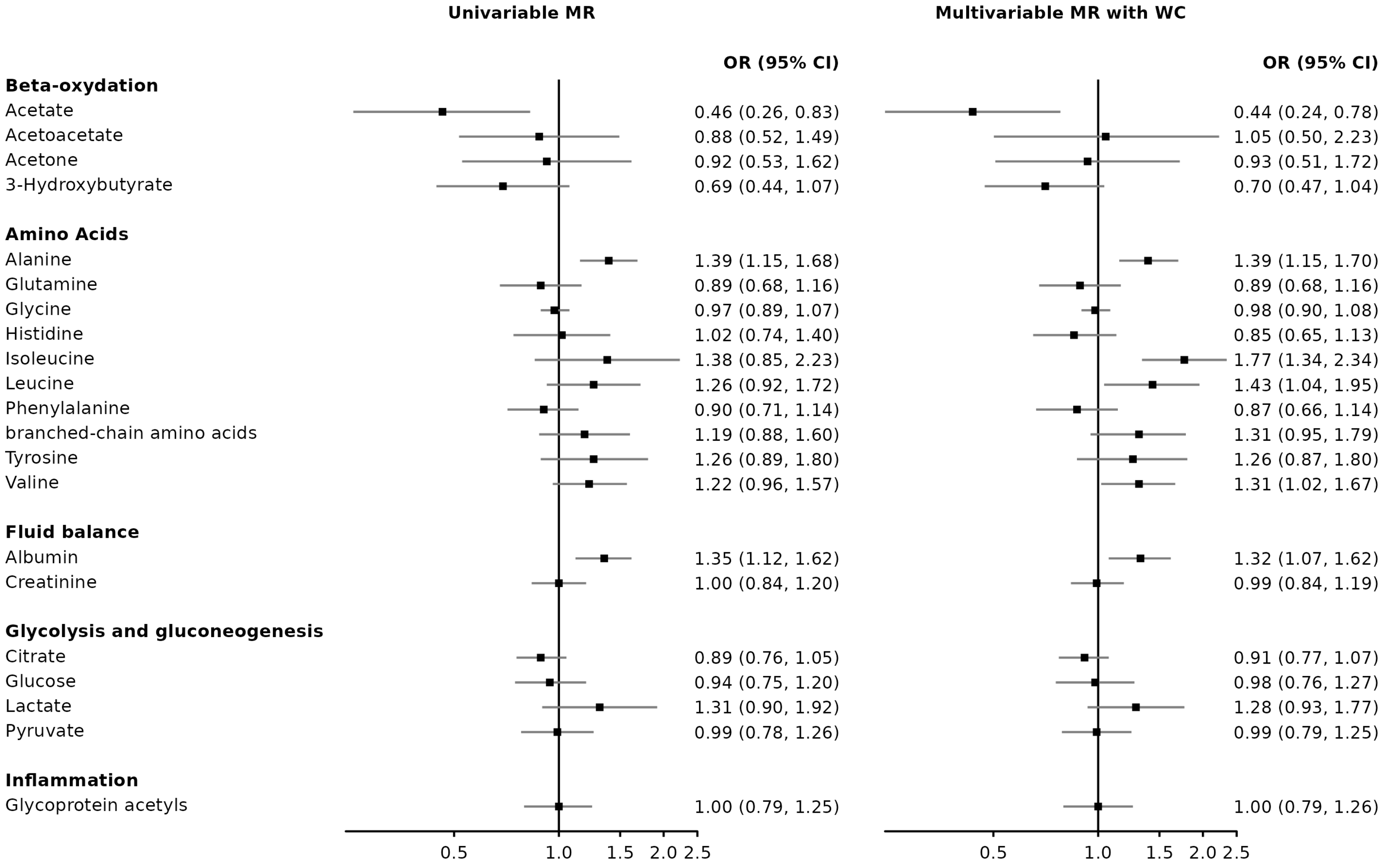

Supplementary Figure 1. Effect of 1 standard deviation increase in metabolites on prevalent NAFLD using univariable MR (UVMR) and multivariable MR correcting for waist circumference (MVMR with WC). Related to STAR methods.

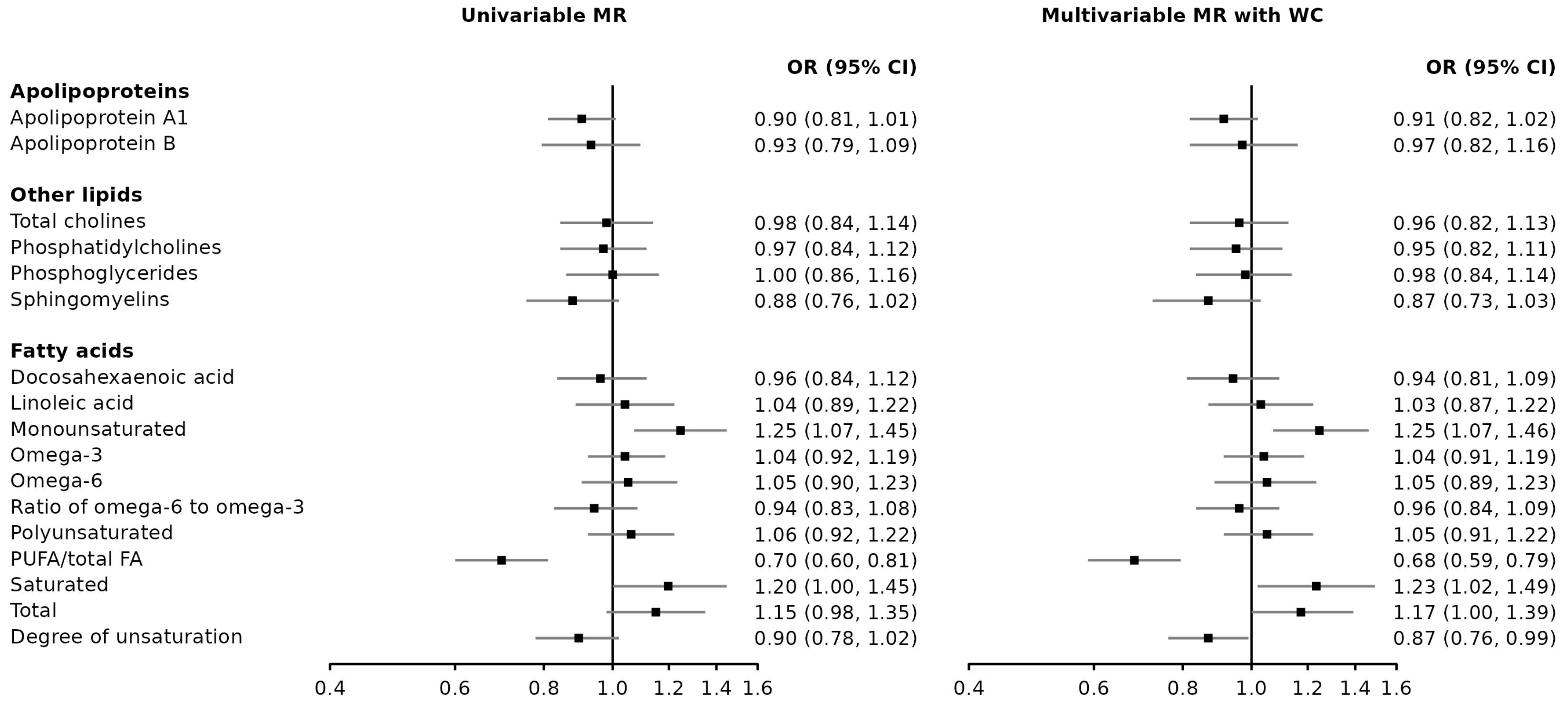

Supplementary Figure 2. Effect of 1 standard deviation increase in lipids on prevalent NAFLD using univariable MR (UVMR) and multivariable MR correcting for waist circumference (MVMR with WC). Related to STAR methods.

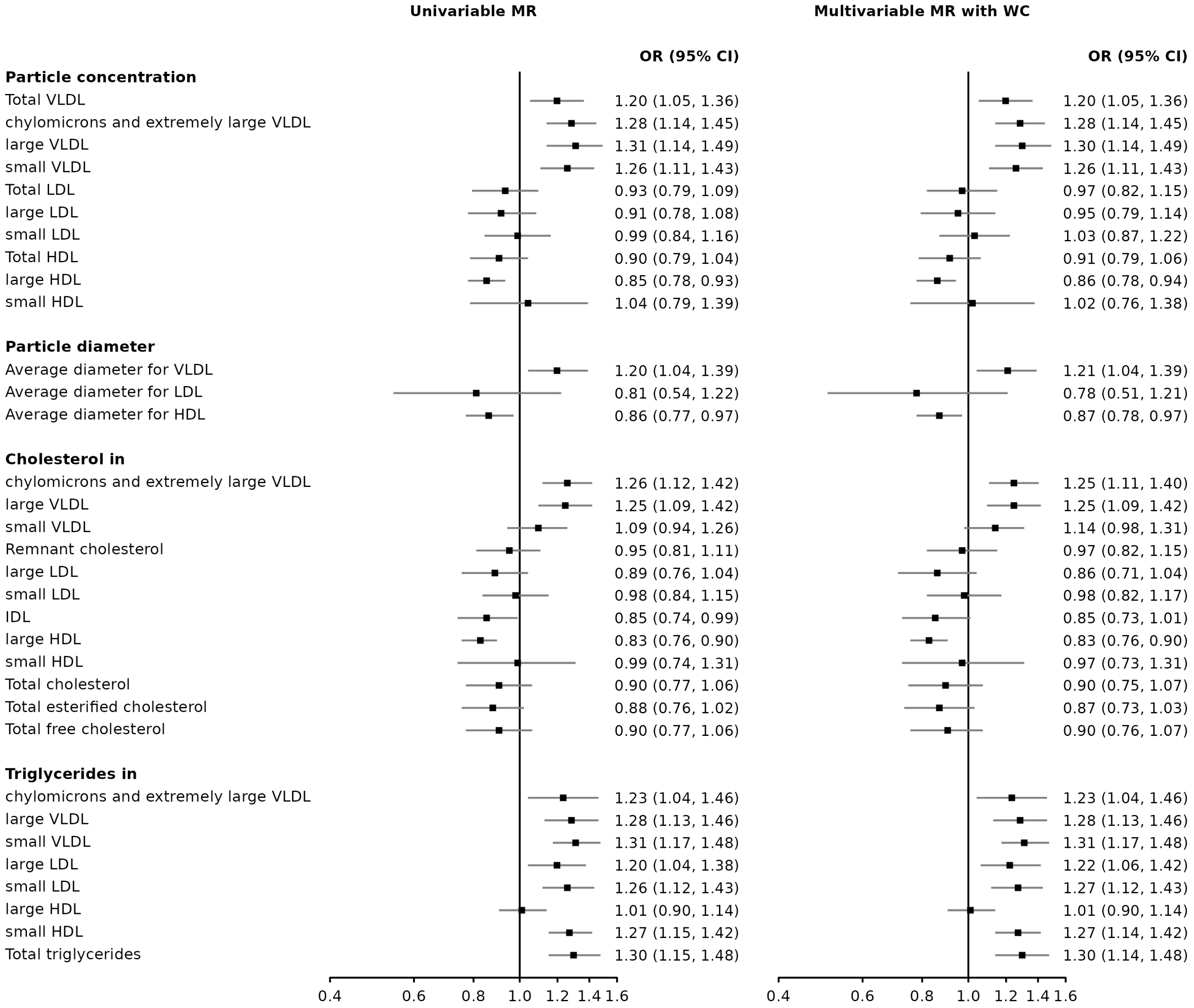

Supplementary Figure 3. Effect of 1 standard deviation increase in lipoproteins on prevalent NAFLD using univariable MR (UVMR) and multivariable MR correcting for waist circumference (MVMR with WC). VLDL : very low density lipoproteins, HDL : high density lipoproteins; LDL : low density lipoproteins. Related to STAR methods.

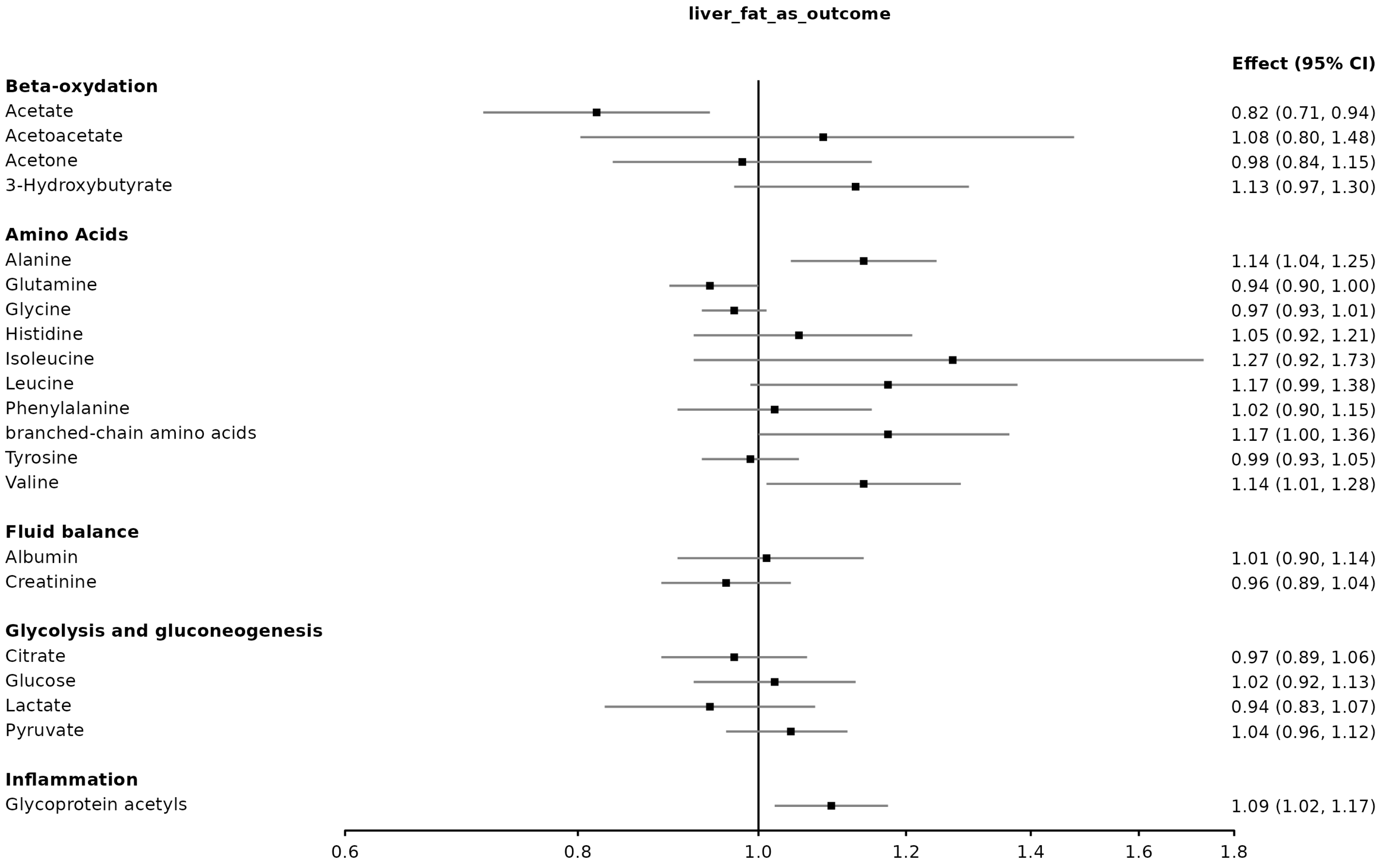

Supplementary Figure 4. Bidirectional MR with liver fat and metabolites. Effect of circulating metabolic factors on liver fat using univariable MR (liver_fat_as_outcome). Effect of liver fat on metabolic factors using univariable MR (liver_fat_as_exposure). Effects are reported on a one standard deviation scale. Related to STAR methods.

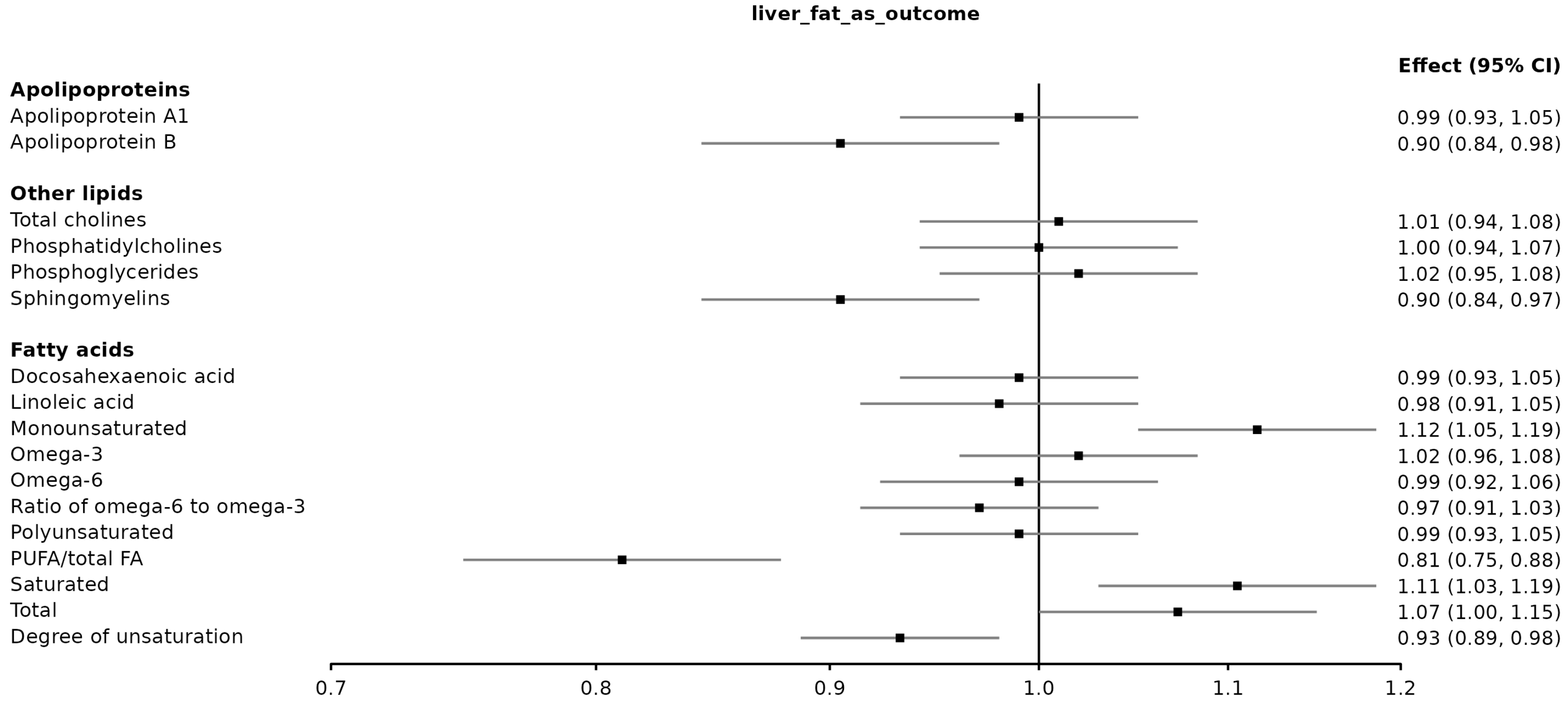

Supplementary Figure 5. Bidirectional MR with liver fat and lipids. Effect of circulating metabolic factors on liver fat using univariable MR (liver_fat_as_outcome). Effect of liver fat on metabolic factors using univariable MR (liver_fat_as_exposure). Effects are reported on a one standard deviation scale. Related to STAR methods.

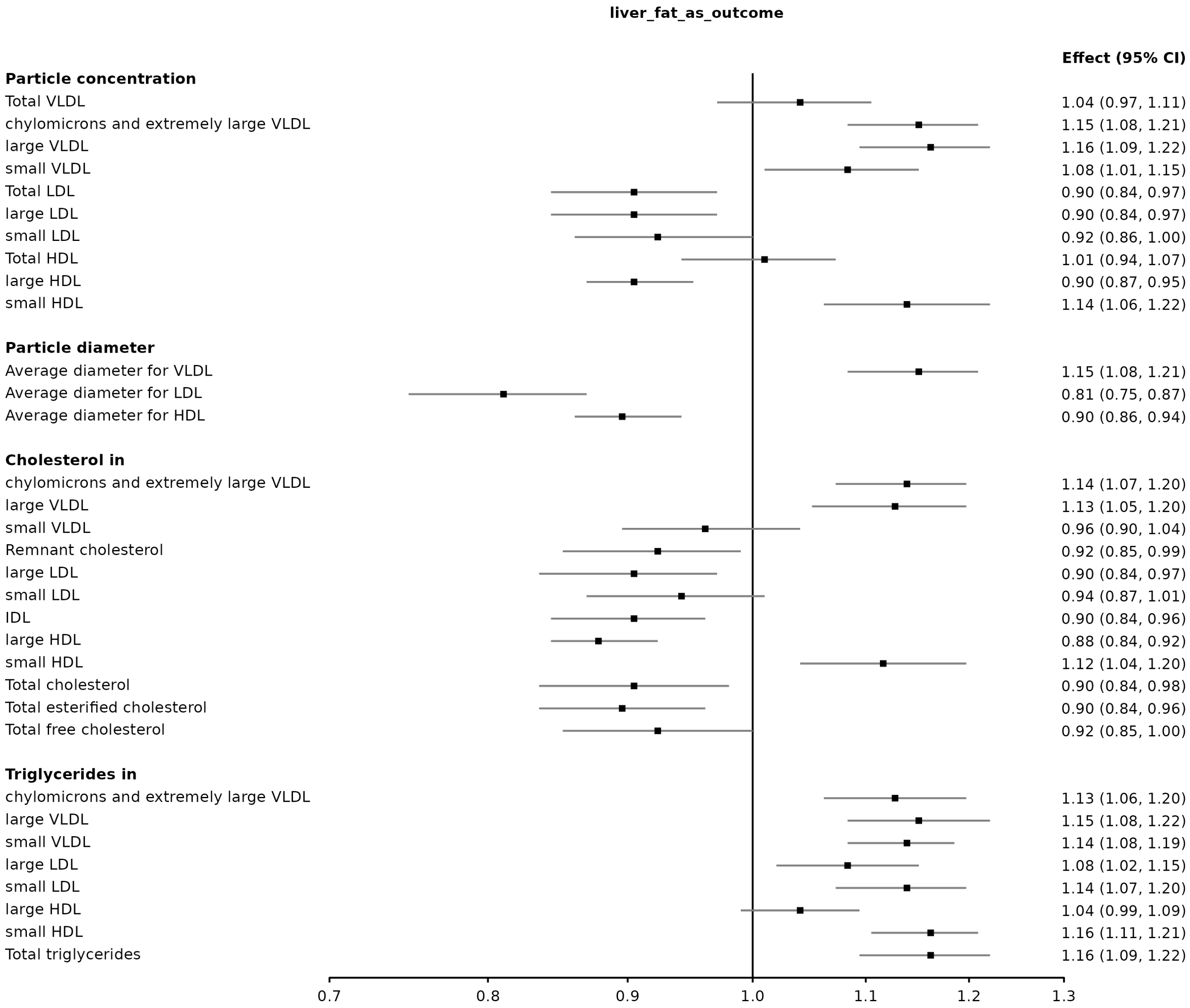

Supplementary Figure 6. Bidirectional MR with liver fat and lipoproteins. Effect of circulating metabolic factors on liver fat using univariable MR (liver_fat_as_outcome). Effect of liver fat on metabolic factors using univariable MR (liver_fat_as_exposure). Effects are reported on a one standard deviation scale. VLDL : very low density lipoproteins, HDL : high density lipoproteins; LDL : low density lipoproteins. Related to STAR methods.

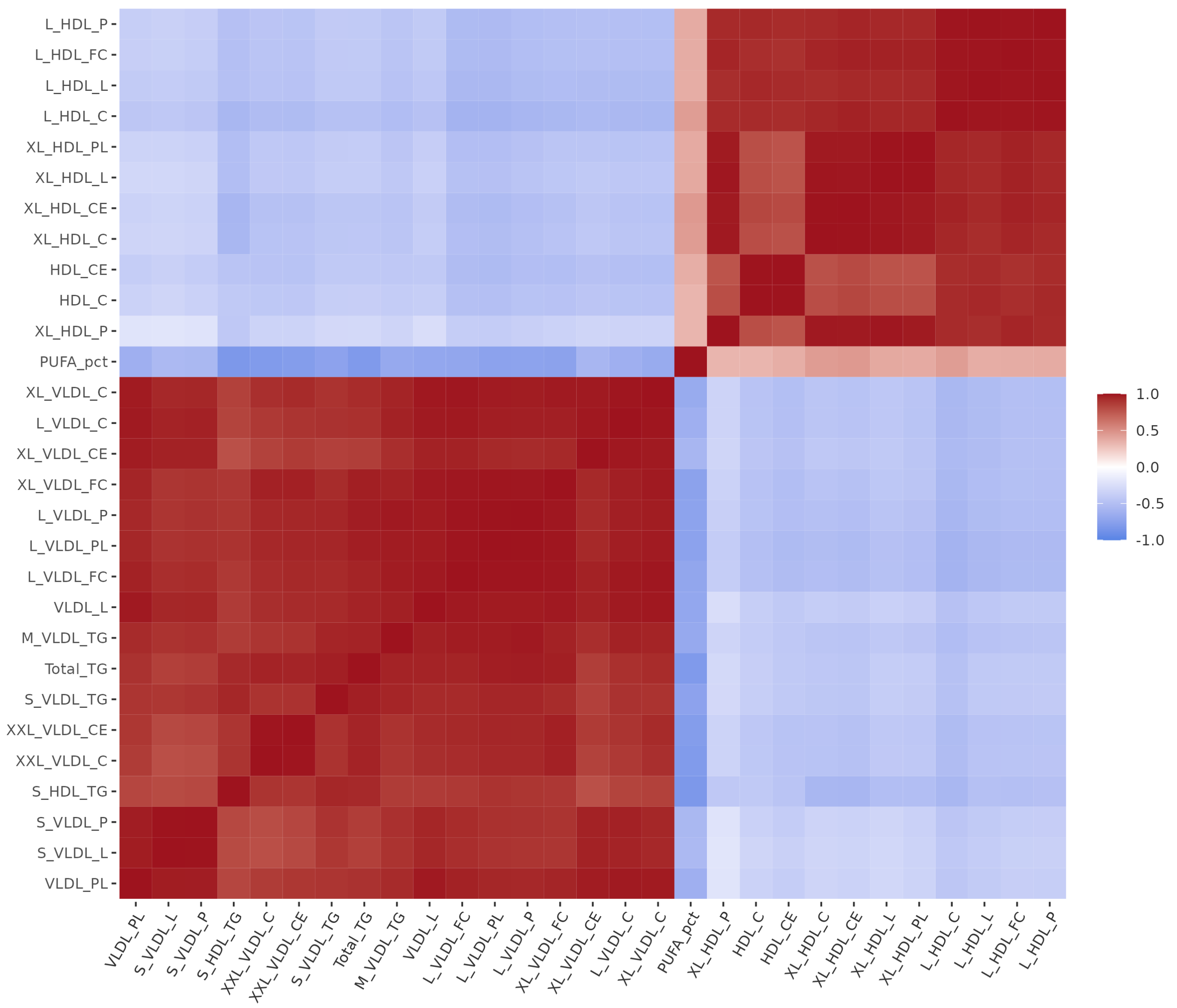

Supplementary Figure 7. Observational correlation matrix clustered with k mean clustering of all causal factors for NAFL-DRelated to STAR methods.
